# Effect of balloon pulmonary angioplasty on cardio-ankle vascular index and biventricular remodeling in patients with chronic thromboembolic pulmonary hypertension

**DOI:** 10.3389/fcvm.2023.1325846

**Published:** 2023-12-01

**Authors:** Shuji Sato, Takuro Ito, Tsuyoshi Tabata, Akihiro Ogawa, Atsuhito Saiki, Kazuhiro Shimizu

**Affiliations:** ^1^ Division of Cardiology, Department of Internal Medicine, Toho University Sakura Medical Center, Chiba, Japan; ^2^ Department of Clinical Functional Physiology, Toho University Sakura Medical Center, Chiba, Japan; ^3^ Department of Rehabilitation, Toho University Sakura Medical Center, Chiba, Japan; ^4^ Center of Diabetes, Endocrine and Metabolism, Toho University Sakura Medical Center, Chiba, Japan

**Keywords:** CTEPH, BPA, CAVI, arterial stiffness, ventricular remodeling

## Abstract

**Background:**

Chronic thromboembolic pulmonary hypertension (CTEPH) is caused by organized pulmonary thrombi, and pulmonary endarterectomy is the only curative treatment. Since balloon pulmonary angioplasty (BPA) has become an established therapeutic option for inoperable CTEPH, prognosis has improved. Recent reports suggest that arterial stiffness evaluated using the cardio-ankle vascular index (CAVI) may play an important role in the cardio-vascular interaction in CTEPH; however, the details remain unclear. This study aimed to clarify the role of CAVI in CTEPH through hemodynamic changes and ventricular remodeling after BPA.

**Methods and results:**

A total of 23 patients with CTEPH who had undergone BPA were enrolled in this study. The mean pulmonary artery pressure (mPAP) and CAVI significantly decreased after BPA [mPAP, 34 (26–45) mmHg to 20 (19–24) mmHg, *p* < 0.0001; CAVI, 9.4 (8.0–10.3) to 8.3 (7.5–9.6), *p* = 0.004]. The echocardiographic right ventricle was significantly decreased, and the left ventricular volume was significantly increased after BPA, indicating significant biventricular remodeling after BPA. Changes in CAVI (ΔCAVI) significantly correlated with changes in mPAP (*r* = 0.45, *p* = 0.03). Additionally, ΔCAVI was significantly correlated with changes in both right ventricular area and left ventricular volume.

**Conclusions:**

Arterial stiffness, evaluated using the CAVI, improved after BPA. Changes in CAVI were significantly correlated with changes in pulmonary arterial pressure and biventricular remodeling. CAVI may play an important role in cardiovascular interactions in patients with CTEPH.

## 1. Introduction

Chronic thromboembolic pulmonary hypertension (CTEPH) is caused by organized thrombi and microvascular remodeling of pulmonary arteries. Patients with CTEPH experience respiratory and/or right-sided heart failure, and their prognoses are extremely poor without appropriate treatment (1). Pulmonary endarterectomy (PEA), a surgical treatment, is the only curative treatment for CTEPH; however, approximately 40% of patients are not candidates for PEA because of surgically inaccessible lesions and comorbidities such as advanced age, malignancy, and congestive heart failure (2). Recently, balloon pulmonary angioplasty (BPA), less invasive catheter interventional therapy, and medical therapy have become standard treatments for patients with inoperable CTEPH (3, 4). As a result, the prognosis of CTEPH has significantly improved even in inoperable patients (5, 6).

Although the prognosis of CTEPH has improved, there are a certain number of patients whose symptoms do not improve sufficiently after BPA (6). One reason for this is thought to be exercise pulmonary hypertension, which is present in nearly half of the patients with CTEPH whose hemodynamics normalize after BPA (7). Moreover, patients with CTEPH with occult left ventricular (LV) disease exist, and occult LV disease may induce exercise pulmonary hypertension (8). However, the precise mechanisms underlying LV dysfunction in patients with CTEPH remain unclear.

Arterial stiffness is one of the most common markers of atherosclerosis and may affect LV function as an afterload. A recent report indicated that arterial stiffness evaluated by pulse wave velocity (PWV) was higher in patients with CTEPH than in healthy controls (9). However, because PWV depends on blood pressure (BP) at the time of measurement (10, 11), it is not suitable for evaluating arterial stiffness in CTEPH, in which hemodynamic conditions, including BP, change during treatment.

The cardio-ankle vascular index (CAVI) is a novel BP-independent arterial stiffness index that can be applied to stiffness parameter β theory (12, 13). The CAVI reflects arterial stiffness from the aortic root to the ankle. In addition, it reflects functional stiffness due to smooth muscle cell contraction or relaxation and plays an important role in hemodynamics by reflecting LV afterload (14–18). Therefore, the CAVI is suitable for evaluating arterial stiffness in patients with CTEPH.

Recently, it was reported that arterial stiffness evaluated by CAVI increased in patients with pulmonary arterial hypertension (PAH), and increased CAVI was associated with poor prognosis (19, 20). These reports suggest that both LV afterload and right ventricular (RV) afterload increase in PAH, potentially deteriorating biventricular remodeling due to increased biventricular afterload. Thus, CAVI may play a significant role in cardiac remodeling in PAH and a similar role in CTEPH, whose hemodynamic status is almost the same as that of PAH, except for organized pulmonary thrombi (21). However, CAVI has rarely been measured in CTEPH, and the precise mechanism underlying cardiovascular interaction in CTEPH has not been clarified.

We report a patient with CTEPH whose CAVI dramatically improved after BPA, along with hemodynamic normalization and biventricular remodeling (22). These findings suggest that changes in arterial stiffness evaluated using CAVI may affect hemodynamic and cardiac remodeling in CTEPH.

This study aimed to investigate the role of CAVI in CTEPH by examining the relationship between changes in CAVI, hemodynamic alterations, and biventricular remodeling after BPA.

## 2. Materials and methods

### 2.1. Patient enrollment and CTEPH diagnosis

We performed BPA on 24 patients with CTEPH between April 2017 and December 2022 at our institution. Of the 24 patients, 23 who completed BPA (1 patient interrupted during treatment) were enrolled in this study, and their clinical data were retrospectively collected. The diagnosis of CTEPH was defined according to the latest European guidelines as follows (23): (1) mean pulmonary arterial pressure (mPAP) >20 mmHg, (2) pulmonary arterial wedge pressure (PAWP) ≤15 mmHg, and (3) organized thrombi detected by enhanced computed tomography (CT) or pulmonary angiography and multiple perfusion defects detected by lung perfusion scintigraphy. CTEPH was diagnosed three months after anticoagulation therapy. Other causes of pulmonary hypertension were ruled out by blood tests, electrocardiography, chest radiography, echocardiography, respiratory functional tests, systemic CT, and right heart catheterization (RHC).

### 2.2. RHC and hemodynamic measurement

All RHC procedures were performed prior to each BPA session. RHC was performed using a 6-Fr wedge pressure catheter. The zero-reference point was set at the mid-thoracic line, and hemodynamic parameters [PAWP, mPAP, and mean right atrial pressure (mRAP)] were measured at the end of expiration. The cardiac output (CO) and cardiac index (CI) were measured using the Fick method. The pulmonary vascular resistance (PVR) was then calculated. BP was measured using a non-invasive method in the catheterization laboratory before RHC. Systolic BP, diastolic BP, and mean BP (mBP) were measured, and systemic vascular resistance (SVR) was calculated.

### 2.3. BPA procedure

All BPA procedures were performed via the common femoral or internal jugular veins. After RHC, an 8-Fr flex sheath was inserted into the main pulmonary artery. Each target pulmonary artery was selected using a 7-Fr or 6-Fr guiding catheter. Detection of organized thrombi using selective pulmonary angiography. In addition, the pressure gradient at each lesion was assessed using a pressure catheter or pressure wire, and all BPAs were performed under pressure guidance (24). For lesion crossing, 0.014 guide wires were used. From 1.2 mm to 7.0 mm balloon catheters were used for lesion dilatation according to vessel diameter and pressure gradient at each lesion. The endpoints of balloon angioplasty were achievement of pulmonary flow grade 3 or an improvement in the pressure ratio (PR) of >0.7 (25). PR was defined as the ratio of distal pulmonary artery pressure (Pd) to proximal pulmonary artery pressure. When the mPAP before BPA was greater than 35 mmHg, the target lesions were carefully dilated such that the Pd did not exceed 35 mmHg to prevent BPA complications, including reperfusion pulmonary edema (24, 25). BPA procedures were repeated until hemodynamic normalization (mPAP < 20 mmHg) or sufficient improvement of symptoms, such as achievement of World Health Organization functional class (WHO-FC) I or II in each patient.

### 2.4. CAVI measurement

All CAVIs were measured on the day before each BPA. After at least 15 min of supine rest, the CAVI was measured using a VaSela VS1500 (Fukuda Denshi Co., Ltd., Tokyo, Japan). The principle of CAVI is described in a previous report (13). Patients with conditions unsuitable for CAVI measurement, such as persistent atrial fibrillation, peripheral artery disease with an ankle-brachial index <0.9, and moderate-to-severe aortic valve stenosis, were not included in this study.

### 2.5. Echocardiography measurement

All echocardiographic examinations were performed on the day before BPA. All echocardiographic examinations were performed using Vivid7, E9, S5, and S6 (GE Healthcare, Boston, MA, USA). Each echocardiographic parameter was analyzed according to the guidelines of the American Society of Echocardiography and the European Association of Cardiovascular Imaging (26).

### 2.6. Timing of the data collection

Hemodynamic parameters measured using the RHC, CAVI, and echocardiographic findings were collected at the time of the first and final BPA in each patient. The following clinical parameters were also collected: WHO-FC, brain natriuretic peptide (BNP), 6-minute walk distance (6 MWD), atrial oxygen saturation (SaO_2_), and mixed oxygen saturation (SvO_2_). The collected parameters were compared before (at the first BPA session) and after BPA (at the final BPA session).

### 2.7. Statistical analysis

Continuous variables were expressed as median [interquartile range (IQR)]. Categorical variables are expressed as numbers (percentages). Continuous variables were compared using the Wilcoxon test, whereas categorical variables were compared using the χ^2^ test. Correlations between changes in the CAVI and other parameters were analyzed using Spearman's correlation coefficient. Statistical significance was set at *p* < 0.05. Statistical analysis was performed using JMP® software version 14.2 (SAS Institute, Cary, NC, United States).

### 2.8. Ethics approval

This study was approved by the Ethics Committee of Toho University Sakura Medical Center (S23019_S22060_S22050_S21060) and was performed in accordance with the Declaration of Helsinki. A comprehensive agreement was obtained from all patients, and the opportunity to refuse participation in this study was provided by an opt-out on our institution's website.

## 3. Results

### 3.1. Patient characteristics before BPA

The patient characteristics before BPA are shown in Table 1. The median patient age was 76 years, and 65.2% of the patients were female. Seventeen patients (73.9%) had a history of APTE. The percentage of patients with hypertension, dyslipidemia, diabetes mellitus, and chronic kidney disease was 39.1%, 69.6%, 30.4%, and 34.8%, respectively. Baseline mPAP was 34 (26–45) mmHg, and median PVR was 6.4 (4.5–12.0) Wood units. The median CO was 3.6 (2.8–4.3) L/min. The median CAVI value was 9.4 (8.0–10.3) before BPA. The median BPA duration was 164 (133–238) days, and the median number of BPA sessions was 5 (4–6) sessions. Home oxygen therapy was required in 14 (60.9%) patients. Approximately half of the patients received warfarin and direct oral anticoagulants. Riociguat was administered to 17 (73.9%) patients prior to BPA.

**Table 1:** Patient characteristics before BPA.

	Patients with CTEPH (*N* = 23)
Age (years)	76 (67–80)
Female	15 (65.2)
BMI (kg/m^2^)	21.4 (19.2–25.1)
History of acute APTE	17 (73.9)
Hypertension	9 (39.1)
Dyslipidemia	16 (69.6)
Diabetes mellitus	7 (30.4)
CKD (>stage 3)	8 (34.8)
mRAP (mmHg)	6 (5–8)
mPAP (mmHg)	34 (26–45)
PAWP (mmHg)	9 (8–14)
PVR (Wood units)	6.5 (4.5–12.0)
CO (L/min)	3.6 (2.8–4.3)
CI (L/min/m^2^)	2.4 (1.8–2.7)
CAVI	9.4 (8.0–10.3)
mBP (mmHg)	95.3 (87.0–102.3)
SVR (Wood units)	26.6 (19.9–29.6)
BPA duration (days)	164 (133–238)
Total BPA session	5 (4–6)
HOT	14 (60.9)
Warfarin	10 (43.5)
DOAC	13 (56.5)
Riociguat	17 (73.9)

Data are presented as median (IQR) or number (%).

BPA, pulmonary angioplasty; BMI, body mass index; APTE, acute pulmonary thromboembolism; CKD, chronic kidney disease; mRAP, mean right atrial pressure; mPAP, mean pulmonary artery pressure; PAWP, pulmonary artery wedge pressure; PVR, pulmonary vascular resistance; CO, cardiac output; CI, cardiac index; CAVI, cardio-ankle vascular index; mBP, mean blood pressure; SVR, systemic vascular resistance; HOT, home oxygen therapy; DOAC, direct oral anticoagulant.

### 3.2. Changes in hemodynamic parameters and CAVI after BPA

**Figure 1:**
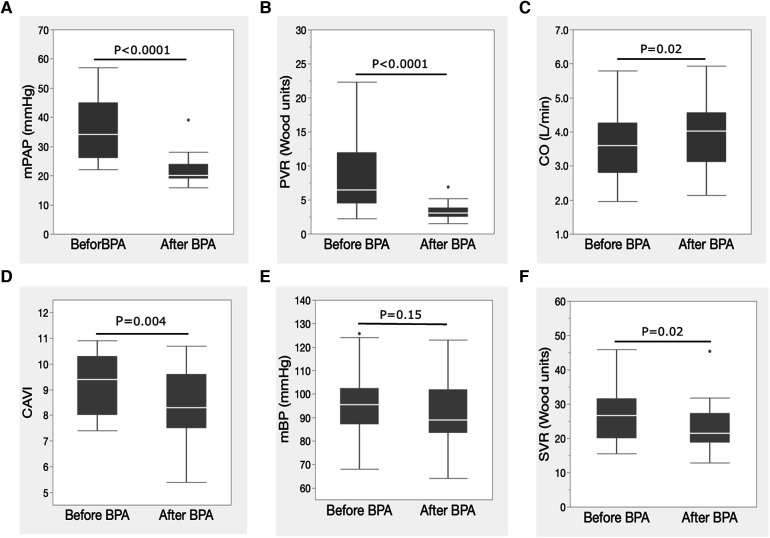
Changes in hemodynamic parameters and CAVI after BPA. (**A**) Changes in mPAP, (**B**) PVR, (**C**) CO, (**D**) CAVI, (**E**) mBP, and (**F**) SVR following BPA. Data are presented as median (interquartile range). White bars, median; boxes, interquartile range; whiskers, ranges excluding statistical outliers; plots, statistical outliers. BPA, pulmonary angioplasty; mPAP, mean pulmonary arterial pressure; PVR, pulmonary arterial resistance; CO, cardiac output; CAVI, cardio-ankle vascular index; mBP, mean blood pressure; SVR, systemic vascular resistance.

The mean PAP and PVR decreased significantly, and the CO significantly increased after BPA (Figures 1A–C). CI also significantly increased after BPA [CI, 2.4 (1.8–2.7) L/min/m^2^ to 2.5 (2.3–2.9) L/min/m^2^, *p* = 0.03]. At the same time, the CAVI decreased significantly (Figure 1D). The mBP levels tended to decrease (Figure 1E), and SVR decreased significantly after BPA (Figure 1F).

### 3.3. Changes in clinical parameters after BPA

The clinical parameters before and after BPA are shown in Table 2. Clinical symptoms were assessed by WHO-FC. The percentage of patients with severe symptoms, such as WHO-FC III or IV, significantly decreased (47.8%–4.3%, *p* = 0.002), and only one patient had severe symptoms after BPA. BNP levels were significantly improved, and 6 MWD were significantly extended after BPA (Table 2). Both SaO_2_ and SvO_2_ levels improved after BPA treatment (Table 2).

**Table 2:** Changes in clinical parameters after BPA.

(*N* = 23)	Before BPA	After BPA	*p*-value
WHO-FC (Ⅰ/Ⅱ/Ⅲ/Ⅳ)	0/12/10/1 (0/52.1/43.4/4.3)	9/13/1/0 (39.1/56.5/4.3/0)	
WHO-FC Ⅲ/Ⅳ	11 (47.8)	1 (4.3)	0.002
BNP (pg/dl)	47.8 (15.8–132.3)	31.3 (15.5–50)	0.01
6MWD (m)	344.5 (298–407.5) (*N* = 22)	392 (315–450.3) (*N* = 22)	0.0008
SaO_2_ (%)	91.8 (89.8–96.0)	94.6 (91.9–95.7)	0.002
SaO_2_ (%)	66.5 (60.9–72.2)	70.8 (67.2–72.4)	0.03

Data are presented as number (%) or median (IQR). The χ^2^ test or Wilcoxon test was used for analysis. WHO-FC, World Health Organization Functional Class; BNP, brain natriuretic peptide; 6 MWD, six-minute walk distance; SaO_2_, arterial oxygen saturation; SVO2, mixed venous oxygen saturation.

### 3.4. Changes in echocardiographic findings after BPA

Table 3 shows the echocardiographic findings before and after BPA. The LV ejection fraction significantly decreased after BPA, but the degree of decrease was slight. Regarding LV diastolic function, the E wave and E/A significantly improved after BPA. The tricuspid regurgitation pressure gradient significantly decreased after BPA. As parameters of RV function, tricuspid annular systolic excursion (TAPSE) and RV fractional change (RVFAC) were significantly improved after BPA.

**Table 3:** Changes in echocardiographic findings after BPA.

(*N* = 23)	Before BPA	After BPA	*p*-value
LVEF (%)	75 (70–76)	68 (66–73)	<0.0001
E (cm/s)	54 (48–71)	68 (59–73)	0.02
A (cm/s)	87 (70–94)	85 (74–97)	0.02
E/A	0.67 (0.56–0.88)	0.74 (0.7–0.86)	0.04
e’ (cm/s)	5.2 (4.7–6.8)	6.1 (5.4–7.2)	0.02
E/e'	9.8 (8.0–12.68)	11.3 (8.1–12.5)	0.79
LAD (mm)	36 (29–42)	36 (33–39)	0.54
LAVI (mL/m2)	22.6 (16.0–29.3)	24.0 (19.0–29.2)	0.13
TRPG	45.5 (35.3–65.0) (*N* = 21)	30.0 (22.5–41.0) (*N* = 21)	<0.0001
TAPSE	16.5 (13.6–18.4)	18.7 (16.7–21.0)	0.01
RVFAC (%)	32.0 (35.0–43.7)	40.0 (34.6–46.0)	0.0004

Data are shown as the median (IQR). The Wilcoxon test was used for the statistical analysis. LVEF, left ventricular ejection fraction; E, peak early diastolic transmitral flow velocity; A, peak atrial systolic transmitral flow velocity; E/A, ratio of E to A; e’, peak early diastolic annular velocity; E/e’, ratio of E to e’; LAD, left atrial diameter; LAVI, left atrial volume index; TRPG, tricuspid regurgitation pressure gradient; TAPSE, tricuspid annular plane systolic excursion; RVFAC, right ventricular fractional area change.

### 3.5. Changes in biventricular remodeling assessed by echocardiography after BPA

**Figure 2:**
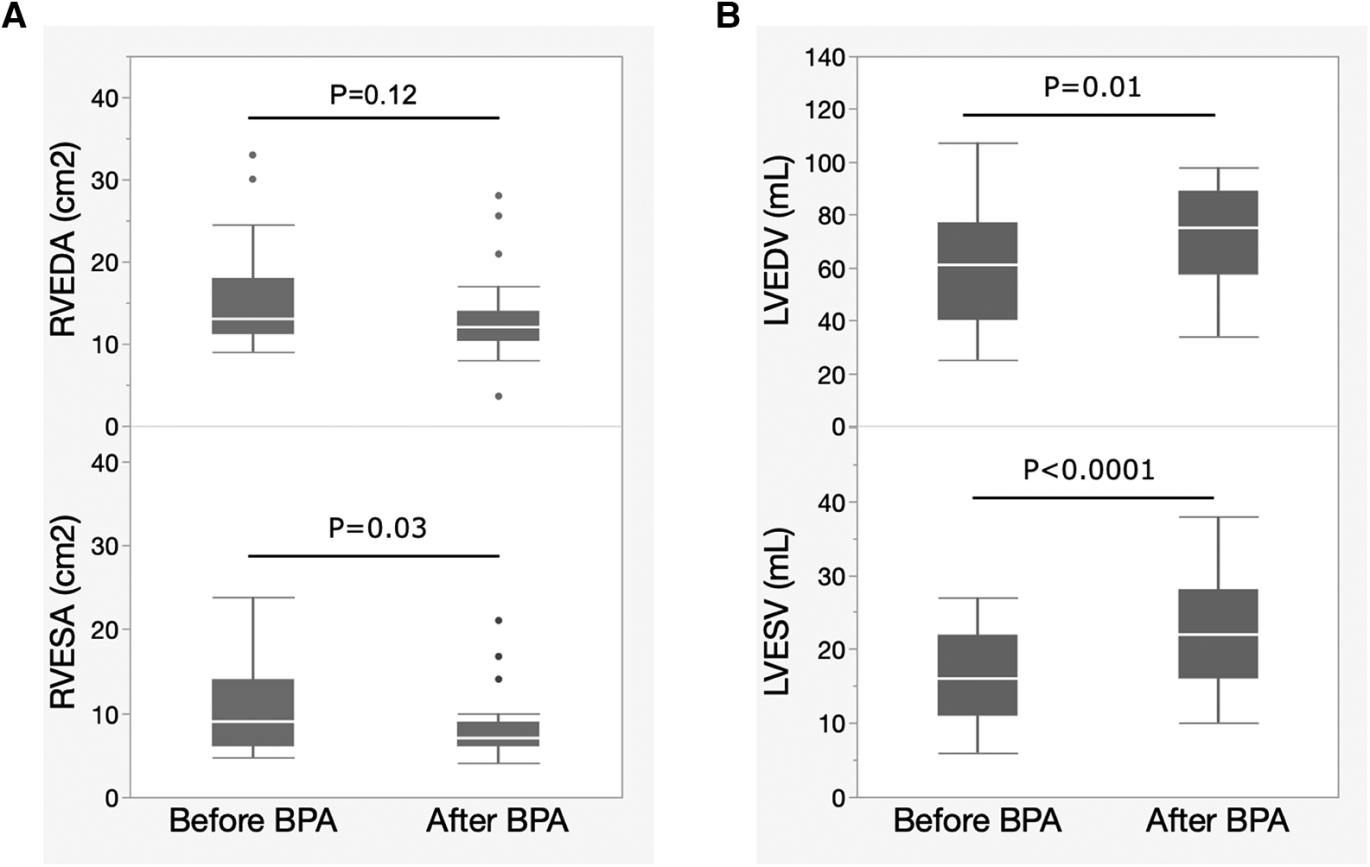
Changes in biventricular remodeling evaluated by echocardiography after BPA. Data are presented as median (interquartile range). White bars, median; boxes, interquartile range; whiskers, ranges excluding statistical outliers; plots, statistical outliers. (**A**) Changes in RVEDA and RVESA levels after BPA exposure. (**B**) Changes in the LVEDV and LVESV after BPA. BPA, balloon pulmonary angioplasty; RVEDA, right ventricular end-diastolic area; RVESA, right ventricular end-systolic area; LVEDV, left ventricular end-diastolic volume; LVESV, left ventricular end-systolic volume.

Figure 2 shows the changes in echocardiographic biventricular remodeling after BPA. RV remodeling was evaluated using the RV end-diastolic area (RVEDA) and the RV end-systolic area (RVESA). LV remodeling was evaluated using the LV end-diastolic volume (LVEDV) and LV end-systolic volume (LVESV). The RVEDA tended to decrease, but the difference was not statistically significant. RVESA significantly decreased after BPA exposure (Figure 2A). LVEDV and LVESV significantly increased after BPA (Figure 2B).

### 3.6. Correlation between changes in CAVI and hemodynamic parameters after BPA

The correlations between changes in CAVI after BPA (ΔCAVI) and changes in hemodynamic parameters or echocardiographic findings are shown in Table 4. The CAVI was significantly correlated with mPAP (*r* = 0.45, *p* = 0.03). Changes in other hemodynamic parameters did not correlate with the CAVI.

**Table 4:** Correlation between changes in CAVI and hemodynamic parameters or echocardiographic findings.

*n* = 23	*r*	*p*-value
Hemodynamic parameters		
ΔmPAP	0.45	0.03
ΔPVR	0.16	0.46
ΔCO	−0.07	0.75
ΔCI	0.09	0.69
ΔmBP	−0.004	0.98
ΔSVR	0.11	0.60
Echocardiographic findings		
ΔLVEF	−0.05	0.81
ΔE	−0.10	0.07
ΔA	0.02	0.93
ΔE/A	−0.23	0.28
Δe'	−0.31	0.16
ΔE/e'	−0.21	0.34
ΔLAD	−0.30	0.16
ΔLAVI	−0.15	0.49
ΔTRPG	0.29	0.21
ΔTAPSE	0.08	0.70
ΔRVFAC	−0.37	0.08
Biventricular remodeling		
ΔRVEDA	0.46	0.03
ΔRVESA	0.59	0.003
ΔLVEDV	−0.44	0.03
ΔLVESV	−0.31	0.16

CAVI, cardio-ankle vascular index; r, Spearman's correlation coefficient; mPAP, mean pulmonary arterial pressure; PVR, pulmonary arterial resistance; CO, cardiac output; CI, cardiac index; mBP, mean blood pressure; SVR, systemic vascular resistance; LVEF, left ventricular ejection fraction; E, peak early diastolic transmitral flow velocity; A, peak atrial systolic transmitral flow velocity; E/A, ratio of E to A; e’, peak early diastolic annular velocity; E/e’, ratio of E to e’; LAD, left atrial diameter; LAVI, left atrial volume index; TRPG, tricuspid regurgitation pressure gradient; TAPSE, tricuspid annular plane systolic excursion; RVFAC, right ventricular fractional area change; RVEDA, right ventricular end-diastolic area; RVESA, right ventricular end-systolic area; LVEDV, left ventricular end-diastolic volume; LVESV, left ventricular end-systolic volume.

### 3.7. Correlation between changes in CAVI and echocardiographic findings after BPA

The correlations between the CAVI and changes in echocardiographic findings after BPA are shown in Table 4. Changes in left ventricular ejection fraction (LVEF) and LV diastolic function (E, A, E/A, e,' and E/e') were not correlated with ΔCAVI. Changes in Tricuspid Regurgitation Peak Gradient (TRPG) and RV function (TAPSE and RVFAC) were not correlated with CAVI.

### 3.8. Correlation between changes in CAVI and biventricular remodeling after BPA

The relationships between CAVI and changes in the RV area (RVEDA and RVESA) and LV volume (LVEDV and LVESV) are shown in Table 4. Both ΔRVEDA and ΔRVESA were positively correlated with ΔCAVI (ΔRVEDA vs. ΔCAVI, *r* = 0.46, *p* = 0.03; ΔRVESA vs. ΔCAVI, *r* = 0.59, *p* = 0.003). ΔLVEDV was negatively correlated with ΔCAVI (*r* = −0.44, *p* = 0.03). LVESV tended to be negatively correlated with CAVI; however, this correlation was not statistically significant.

## 4. Discussion

This study is the first to evaluate whether CAVI is associated with hemodynamic and biventricular remodeling in CTEPH. After BPA, mPAP, and PVR improved to near the normal range, and the main clinical parameters, such as clinical symptoms assessed by WHO-FC, exercise tolerance, and oxygenation, also improved. The echocardiographic LV diastolic function and RV function (TAPSE and RVFAC) improved, and significant biventricular remodeling was observed after BPA. CAVI significantly improved after BPA, especially in association with changes in pulmonary arterial pressure and biventricular remodeling.

### 4.1. Mechanisms of CAVI improvement after BPA

Tatebe et al. reported that CAVI improved after BPA along with the improvement of multiple metabolic disorders. However, the mechanisms underlying CAVI improvement have not yet been elucidated (27). Generally, CAVI improvements are thought to be due to the regression of atherosclerosis; therefore, CAVI improvement after BPA in CTEPH is a remarkable phenomenon. BPA essentially improves hemodynamics by dilating the pulmonary arteries; therefore, we should focus on the functional changes of CAVI according to hemodynamic changes rather than structural atherosclerosis regression. Considering these functional changes, CAVI may provide new insights from the viewpoint of cardiovascular interactions.

Radchenko et al. reported that the CAVI was higher in patients with PAH than in healthy controls (19). In the present study, the median CAVI value in CTEPH was 9.4, which was slightly higher than that of healthy Japanese subjects (males, 9.35 ± 1.0; females, 8.71 ± 0.74, 70–75 years old) (28). Although statistical analysis could not be performed, CAVI may increase in patients with CTEPH, similar to PAH. Furthermore, the improvement in CAVI after BPA also indicated that CTEPH showed a high CAVI and that arterial stiffness was enhanced in CTEPH. Radchenko et al. suggested that systemic inflammation of the arteries might preexist in cases of PAH, resulting in increased CAVI (19, 20). However, it is unclear whether the increased CAVI is due to structural atherosclerosis progression or functional stiffness enhancement, which reflects smooth muscle cell contraction. However, the improvement in CAVI after BPA in this study strongly suggests that the enhanced CAVI in CTEPH may be due to functional stiffness enhancement with arterial smooth muscle contraction. Furthermore, a decrease in mBP (not significant) and SVR (Figures 1E,F) supported that CAVI improvement was predominantly due to relief of smooth muscle contraction accompanied by improvement of pulmonary hypertension after BPA rather than atherosclerosis regression or suppression of systemic inflammation.

Activation of the sympathetic nervous system and neurohormonal factors is assumed to cause smooth muscle contraction in CTEPH (29, 30). In addition, chronic thromboembolic pulmonary arteries may produce vasocontraction-inducing factors, such as endothelin, or inducing factors, such as catecholamines, angiotensin, and vasopressin. Currently, there is no data or evidence to discuss this problem. Further studies are required to confirm this hypothesis.

In any case, a high CAVI in CTEPH might further worsen hemodynamics by increasing the LV afterload. Both RV and LV afterloads are increasing in CTEPH, which may lead to a vicious hemodynamic cycle in patients with CTEPH, leading to a poor prognosis.

### 4.2. Relationship between CAVI and biventricular remodeling

Pulmonary hypertension leads to significant cardiac remodeling in the right heart system due to increased PVR and pressure (21, 23). An enlarged RV compresses the LV cavity, and LV filling decreases because of pulmonary circulation disturbances. Therefore, LV remodeling progresses with RV remodeling according to the severity of pulmonary hypertension. Moreover, increased CAVI may lead to further progression of LV remodeling by increasing the LV afterload (22).

Kishiki et al. reported that LV dysfunction in PAH evaluated by LV strain was observed not only in septal segments but also in non-septal segments, suggesting that unknown mechanisms may impair LV function in addition to the interventricular interaction caused by the enlarged RV (31). We also encountered patients with CTEPH whose LV function, as evaluated by LV strain, improved after administration of riociguat, a guanylate cyclase stimulator (32). In these cases, LV dysfunction was observed in both the septal and non-septal LV segments and improved with an improvement in CAVI. These findings suggest that increased CAVI may be one of the reasons that LV function is impaired by increasing LV afterload in pulmonary hypertension; however, further studies are needed to clarify the details.

**Figure 3:**
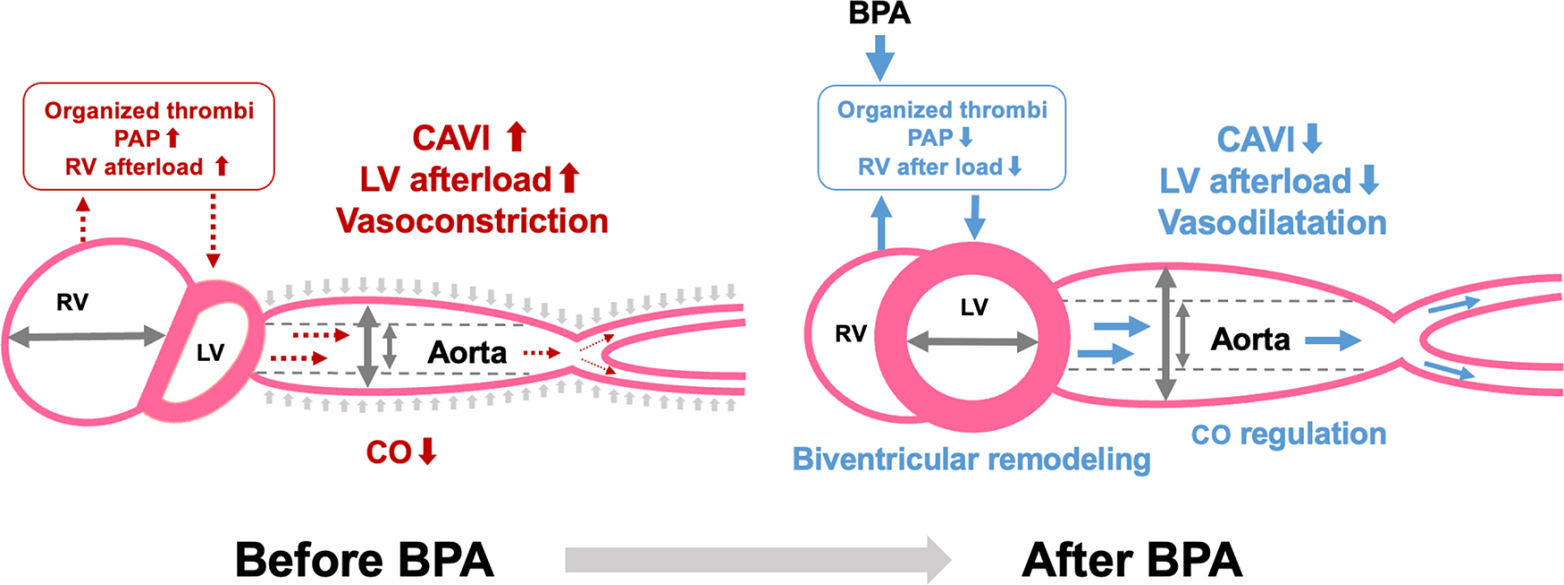
Relationship between CAVI and biventricular remodeling in CTEPH. CAVI, cardio-ankle vascular index; BPA, balloon pulmonary angioplasty; PAP, pulmonary arterial pressure; RV, right ventricle; LV, left ventricle; CO, cardiac output.

A schematic representation of the relationship between CAVI and biventricular remodeling in CTEPH is shown in Figure 3. Similar to PAH, significant biventricular remodeling, observed as an enlarged RV and an oppressed LV, occurs in CTEPH. In addition, increased CAVI may further enhance LV oppression by increasing the LV afterload. The organized thrombi were dilated and the entire pulmonary blood flow dramatically improved after several BPA sessions. Consequently, the RV afterload is reduced, leading to negative RV remodeling. Simultaneously, LV compression is released, and LV filling is increased, resulting in LV-positive remodeling. Moreover, decreased CAVI after BPA may accelerate LV-positive remodeling by reducing the LV afterload. Thus, CAVI is closely related to biventricular remodeling.

While there were strong correlations between ΔCAVI and biventricular remodeling, ΔCO did not show a correlation with ΔCAVI. Systemic vessels contract or relax in response to hemodynamics to maintain CO, leading to constant changes in vessel tone. This variability may explain why changes in CAVI do not always align with changes in CO. Conducting shorter-term observations may provide a clearer understanding of the relationship between CAVI and CO.

### 4.3. Clinical implications of CAVI in CTEPH treatment

CAVI has been reported to play a prognostic role in PAH (20); however, it is unclear whether CAVI is a prognostic marker in CTEPH. Mean PAP is one of the most well-known prognostic markers of CTEPH; therefore, it is important to evaluate mPAP during CTEPH treatment (1). However, invasive RHC is required to accurately evaluate the mPAP. CAVI is a non-invasive measurement method with high reproducibility (12). Given the significant correlation observed between changes in CAVI and changes in mPAP in this study, CAVI may be a non-invasive index to estimate treatment effects during BPA, thus potentially emerging as a new treatment target for CTEPH. In addition, pulmonary vasodilators such as riociguat may decrease CAVI by dilating both the pulmonary and systemic arteries, similar to BPA. A multimodal approach to CTEPH may contribute to further CAVI-lowering treatment and lead to further improvement in pulmonary hypertension (33, 34).

In this study, we have not investigated long-term course of CAVI after BPA. In our case report, CAVI value decreased significantly from 10.0 to 5.8 at 6 months after the final BPA session, while maintaining hemodynamic normalization (22). Therefore, a low CAVI value may indicate hemodynamic stabilization in patients with CTEPH even in the long term. Further studies are needed to elucidate the role of CAVI in the long term after BPA.

The precise mechanisms underlying CAVI improvement in patients with CTEPH remain unclear. However, the CAVI-lowering CTEPH treatment may lead to a better prognosis. By evaluating CAVI, we can assess systemic vascular function in addition to hemodynamics and cardiac function, thereby contributing to a better understanding of the pathophysiology of CTEPH in terms of cardiovascular interactions (35).

### 4.4. Study limitations

First, this was a retrospective study with a small sample size. Therefore, a large-scale prospective study is required to clarify the role of CAVI in the treatment of CTEPH. Second, in this study, 73.9% of the patients received riociguat before BPA. Riociguat may decrease the CAVI, reflecting its vasodilatory effect.

## 5. Conclusions

In this study, CAVI in patients with CTEPH improved after BPA, along with hemodynamic improvement and biventricular remodeling. Changes in CAVI were significantly correlated with changes in pulmonary arterial pressure and biventricular remodeling. CAVI may play an important role in the pathophysiology of CTEPH through biventricular remodeling. Evaluating CAVI during BPA may lead to a better understanding of the cardiovascular interactions in CTEPH.

## Data Availability

The raw data supporting the conclusions of this article will be made available by the authors, without undue reservation.
