# Corrigendum: Effect of balloon pulmonary angioplasty on cardio-ankle vascular index and biventricular remodeling in patients with chronic thromboembolic pulmonary hypertension

**DOI:** 10.3389/fcvm.2024.1377556

**Published:** 2024-02-07

**Authors:** Shuji Sato, Takuro Ito, Tsuyoshi Tabata, Akihiro Ogawa, Atsuhito Saiki, Kazuhiro Shimizu

**Affiliations:** ^1^ Division of Cardiology, Department of Internal Medicine, Toho University Sakura Medical Center, Chiba, Japan; ^2^ Department of Clinical Functional Physiology, Toho University Sakura Medical Center, Chiba, Japan; ^3^ Department of Rehabilitation, Toho University Sakura Medical Center, Chiba, Japan; ^4^ Center of Diabetes, Endocrine and Metabolism, Toho University Sakura Medical Center, Chiba, Japan

**Keywords:** CTEPH, BPA, CAVI, arterial stiffness, ventricular remodeling

A Corrigendum on Effect of balloon pulmonary angioplasty on cardio-ankle vascular index and biventricular remodeling in patients with chronic thromboembolic pulmonary hypertension By Sato S, Ito T, Tabata T, Ogawa A, Saiki A and Shimizu K (2023) Front. Cardiovasc. Med. 10:1325846. doi: 10.3389/fcvm.2023.1325846

In the published article, there was an error in the Ethics statement. Since this article is a retrospective study by an opt out on our institution's website as shown in the materials and methods section, we did not obtain written informed consent. The original Ethic Statement, “The studies involving humans were approved by Ethics Committee of Toho University Sakura Medical Center. The studies were conducted in accordance with the local legislation and institutional requirements. The participants provided their written informed consent to participate in this study”, has been changed. The last sentence, “The participants provided their written informed consent to participate in this study”, is incorrect. The corrected Ethics Statement appears below.

Ethics Statement

The studies involving humans were approved by Ethics Committee of Toho University Sakura Medical Center. The studies were conducted in accordance with the local legislation and institutional requirements.

The authors apologize for this error and state that this does not change the scientific conclusions of the article in any way. The original article has been updated.

